# Gepulste DPOAE in Verlaufsmessungen: Kombiniertes Analyseparadigma von gleichzeitig auftretenden Veränderungen in Hörschwellen und DPOAE

**DOI:** 10.1007/s00106-024-01477-0

**Published:** 2024-05-27

**Authors:** Katharina Bader, Dennis Zelle, Anthony W. Gummer, Ernst Dalhoff

**Affiliations:** 1 Klinik für Hals-Nasen-Ohren-Heilkunde, Universitätsklinikum Tübingen, Elfriede-Aulhorn-Straße 5, 72076 Tübingen, Deutschland; 2 Sektion für Physiologische Akustik und Kommunikation, Universitäts-HNO-Klinik Tübingen, Tübingen, Deutschland; 3 Earlab GmbH, Tübingen, Deutschland

**Keywords:** Analyse im Zeitbereich, Cochleärer Verstärker, Ototoxizität, Test-Retest-Zuverlässigkeit, Time domain analysis, Cochlea amplifier, Ototoxicity, Test-retest reliability

## Abstract

**Hintergrund:**

Bisher gibt es keinen Konsens darüber, wie Ototoxizität in Verlaufsmessungen standardisiert zu erfassen ist. Für die Diagnostik von Schädigungen des cochleären Verstärkers sind Messverfahren notwendig, die eine möglichst hohe Test-Retest-Zuverlässigkeit und eine hohe Aussagekraft hinsichtlich persistierender Schädigungen aufweisen. Hörschwellenschätzungen auf der Grundlage von Kurzpuls-DPOAE-Pegelkarten („estimated distortion-product thresholds“, *L*_EDPT_) berücksichtigen individuell optimale DPOAE-Anregungspegel und erlauben eine zuverlässige quantitative Schätzung des cochleär bedingten Hörverlusts.

**Methodik:**

Hörschwellen wurden mithilfe von *L*_EDPT_ objektiv geschätzt und mit einer modifizierten Békésy-Tracking-Audiometrie (*L*_TA_) subjektiv erfasst. Die Messungen wurden siebenmal innerhalb von drei Monaten bei 14 Frequenzen (*f*_2_ = 1–14 kHz) in 20 Ohren (PTA_4 (0,5–4_ _kHz)_ < 20 dB HL) durchgeführt. Die Rekonstruktion des DPOAE-Wachstumsverhaltens in Abhängigkeit von den Anregungspegeln *L*_1_,*L*_2_ erfolgte auf der Grundlage von 21 DPOAE-Amplituden und ermöglichte mithilfe einer numerischen Anpassung einer nichtlinearen mathematischen Funktion die Berechnung eines *L*_EDPT_ für jede Anregungsfrequenz. Für die gleichzeitige kombinierte Betrachtung wurden Verteilungen der Hörschwellen (*L*_TA_, *L*_EDPT_), der DPOAE-Pegel (*L*_DP_) und Kombinationen davon ermittelt.

**Ergebnisse:**

Einzeln betrachtet wiesen *L*_TA_ und *L*_EDPT_ jeweils eine Test-Retest-Zuverlässigkeit mit einem Median der absoluten Differenzen (AD) von 3,2 dB bzw. 3,3 dB auf, der sich durch Anwendung eines kombinierten Analyseparadigmas aus *L*_EDPT_, *L*_DP_ und *L*_TA_ auf 2,0 dB signifikant reduzieren ließ.

**Schlussfolgerung:**

Es ist zu erwarten, dass ein auf einer Kombination von *L*_EDPT_, überschwelligen *L*_DP_, und feinstrukturreduzierter *L*_TA_ basierendes Analyseparadigma eine höhere Güte (Sensitivität und Spezifität) des Tests erzielt, um pathologische oder auch regenerative Veränderungen der äußeren Haarsinneszellen zuverlässig zu detektieren.

## Abstract (en)

**Background:**

To date, there is no consensus on how to standardize the assessment of ototoxicity in serial measurements. For the diagnosis of damage to the cochlear amplifier, measurement methods are required that have the highest possible test-retest reliability and validity for detecting persistent damage. Estimated distortion-product thresholds (*L*_EDPT_) based on short-pulse distortion-product otoacoustic emission (DPOAE) level maps use individually optimal DPOAE stimulus levels and allow reliable quantitative estimation of cochlea-related hearing loss.

**Materials and methods:**

Hearing thresholds were estimated objectively using *L*_EDPT_ and subjectively using modified Békésy tracking audiometry (*L*_TA_). Recordings were performed seven times within three months at 14 frequencies (*f*_2_ = 1–14 kHz) in 20 ears (PTA_4__(0.5–4_ _kHz)_ < 20 dB HL). Reconstruction of the DPOAE growth behavior as a function of the stimulus levels *L*_1_, *L*_2_ was performed on the basis of 21 DPOAE amplitudes. A numerical fit of a nonlinear mathematical function to the three-dimensional DPOAE growth function yielded *L*_EDPT_ for each stimulus frequency. For the combined analysis, probability distributions of hearing thresholds (*L*_TA_, *L*_EDPT_), DPOAE levels (*L*_DP_), and combinations thereof were determined.

**Results:**

*L*_TA_ and *L*_EDPT_ each exhibited a test-retest reliability with a median of absolute differences (AD) of 3.2 dB and 3.3 dB, respectively. Combining *L*_EDPT_, *L*_DP_, and *L*_TA_ into a single parameter yielded a significantly smaller median AD of 2.0 dB.

**Conclusion:**

It is expected that an analysis paradigm based on a combination of *L*_EDPT_, suprathreshold *L*_DP_, and fine-structure-reduced *L*_TA_ would achieve higher test performance (sensitivity and specificity), allowing reliable detection of pathological or regenerative changes in the outer hair cells.

Bisher gibt es keinen Konsens darüber, wie Ototoxizität in Verlaufsmessungen standardisiert zu erfassen ist. Gegenwärtig in der Klinik eingesetzte DPOAE unterscheiden weder die beiden DPOAE-Komponenten infolge ihrer kontinuierlichen Anregung, noch berücksichtigen sie die individuelle Mittelohrübertragungsfunktion. Gepulste DPOAE mit individuell optimalen Anregungspegeln führen bei normalhörenden Probanden zu einer Verbesserung der Aussagekraft und einer geringeren Variabilität. Eine kombinierte Analyse von gepulsten DPOAE und Hörschwellen löst Änderungen des Hörstatus am besten auf.

## Hintergrund und Fragestellung

Das Ziel von Verlaufsmessungen der Funktionsfähigkeit des cochleären Verstärkers ist es, tatsächlich auftretende Veränderungen mit hoher Sensitivität und Spezifität abzubilden. Im klinischen Alltag dienen Verlaufsmessungen beispielsweise dazu, rechtzeitig den Einfluss ototoxischer Substanzen auf die Funktionsfähigkeit des Gehörs oder den Effekt regenerativer Therapieansätze zu erkennen. Bisher gibt es allerdings keinen internationalen Konsens darüber, wie Ototoxizität oder Regeneration standardisiert zu erfassen ist. Die American Academy of Audiology bewertet die Bestimmung der Reintonhörschwelle vor allem im hochfrequenten Bereich sowie die Messung von Distorsionsprodukt-otoakustischen Emissionen (DPOAE) als die zuverlässigsten klinisch anwendbaren Methoden [10].

DPOAE entstehen durch Intermodulation infolge der simultanen Anregung der Cochlea mit zwei Anregungstönen der Frequenzen *f*_1_ und *f*_2_ (typisch, *f*_2_*/f*_1_ *≈* 1,2) und Anregungspegeln *L*_1_ und *L*_2_. DPOAE basieren direkt auf der Nichtlinearität der mechanoelektrischen Transduktion der äußeren Haarsinneszellen in einem begrenzten Bereich um den cochleären Abbildungsort der Anregungsfrequenz *f*_2_ und liefern dadurch frequenzspezifische Informationen über die Funktionalität des cochleären Verstärkers [2].

Eine bisherige Empfehlung zu Monitoring und Evaluation der Ototoxizität bei Kindern und Jugendlichen beinhaltet die Anamnese, die Reintonaudiometrie für die Frequenzen 1–8 kHz, DPOAE und die Tympanometrie [6]. Demnach sollte eine Testbatterie verschiedener Methoden durchgeführt werden, da einzelne Methoden nicht hinreichend aufschlussreich sind. Anhand von Studien mittlerer Evidenz detektieren DPOAE Veränderungen des Hörvermögens früher als die Reintonaudiometrie und weisen eine höhere Sensitivität gegenüber subtilen oder subklinischen Veränderungen auf [7]. DPOAE-Schwellen zeigten in zwei Studien eine höhere Empfindlichkeit als einzelne DPOAE-Pegel [13, 22]. Eine Hochfrequenz-Audiometrie (HFA) bei 9–16 kHz kann Hörveränderungen häufiger als eine Reintonaudiometrie detektieren [1]. Bei Kindern werden DPOAE verwendet, um frühzeitig ototoxische, cisplatin-bedingte Abnahmen der Amplitude oder des Signal-Rausch-Abstands (SNR) der DPOAE aufzudecken [14].

In audiologischen Verlaufsuntersuchungen ist eine hohe Test-Retest-Zuverlässigkeit des verwendeten Messverfahrens essenziell, um systematische pathologisch oder regenerativ bedingte Veränderungen von zufälligen Messungenauigkeiten abzugrenzen, wobei die Aussagekraft des Messverfahrens über den Zustand des Untersuchungsobjekts ebenso bedeutsam ist. So können beispielsweise Pegeländerungen (< 6 dB) klinisch gebräuchlicher DPOAE alleine eine mittels Reintonaudiometrie verifizierte ototoxische Hörschwellenerhöhung nicht mit ausreichender Sensitivität und Spezifität vorhersagen [16]. Multivariate Analysen, die DPOAE-Pegel bei benachbarten Frequenzen, SNR und eine Dosis-Wirkungs-Beziehung berücksichtigen, erhöhen zwar die Vorhersagekraft, um eine ototoxische Hörschädigung zu detektieren, konnten sich allerdings bisher im klinischen Alltag nicht durchsetzen [16]. Folglich gibt es bisher keine klinisch validierte, signifikante DPOAE-Änderung, die einen potenziellen cochleären Schaden vorhersagt [15, 22].

DPOAE werden aktuell in der Klinik als nützliche, ergänzende Untersuchung zur Diagnostik des Innenohrzustands angesehen, die aber Grenzen in ihrer Aussagekraft aufweisen [11]. Hierfür gibt es drei Hauptursachen: 1. DPOAE bestehen im Wesentlichen aus zwei Komponenten, der nichtlinearen Distorsionskomponente und der kohärenten Reflexionskomponente, die an unterschiedlichen Orten entlang der Basilarmembran durch unterschiedliche Mechanismen entstehen [25]. Abhängig vom relativen Pegel- und Phasenverhältnis zwischen den Komponenten interferieren die Wellen, und können damit zu artefaktbehafteten Messergebnissen führen [29]. 2. DPOAE-Signale sind insbesondere hinsichtlich der retrograden Übertragung von den individuellen Mittelohreigenschaften beeinflusst [17]. 3. DPOAE-Pegel zeigen eine relativ begrenzte Korrelation mit dem cochleär bedingten Hörverlust, wobei die Beziehung sowohl vom Pegel als auch von der Frequenz nichtlinear abhängig ist [4, 12].

Eine erweiterte DPOAE-Diagnostik stellen DPOAE-Wachstumsfunktionen dar, die die DPOAE-Amplitude in der linearen Einheit des Schalldrucks als Funktion des Anregungspegels *L*_2_ für jede Frequenz semilogarithmisch abbilden. Durch Extrapolation einer Regressionsgerade wird eine Distorsionsproduktschwelle geschätzt („estimated distortion-product threshold“, EDPT), die annähernd im Verhältnis 1:1 mit der cochleär bedingten Hörschwelle korreliert [5]. Die diagnostische Präzision verbessert sich erheblich durch die artefaktfreie Erfassung und Analyse von DPOAE im Zeitbereich mithilfe gepulster Stimuli [8, 27, 29, 30] sowie der Anwendung individuell optimaler, frequenzspezifischer Anregungspegel, die mithilfe DPOAE-Pegelkarten erfasst werden [28]. DPOAE-Pegelkarten bilden das DPOAE-Wachstumsverhalten in Abhängigkeit von Anregungspegelkombinationen ab, die eine erweiterte Fläche im *L*_1_,*L*_2_-Raum abtasten, und ermöglichen mithilfe einer numerischen Anpassung einer nichtlinearen mathematischen Funktion die Berechnung einer geschätzten Distorsionsproduktschwelle *L*_EDPT_, ohne davon abhängig zu sein, dass ein a priori gewählter Anregungspfad den individuellen Gegebenheiten nahekommt [28].

*L*_EDPT_ stellen eine vielversprechende Methode dar, um tatsächlich auftretende Veränderungen des cochleären Verstärkers mit hoher Sensitivität und Spezifität in Verlaufsuntersuchungen abzubilden, da sie eine Hörminderung mit hoher Genauigkeit quantifizieren können [28] und eine hohe Test-Retest-Zuverlässigkeit aufweisen [3]. DPOAE-Pegel, die in einschlägigen Arbeiten gegenüber DPOAE-Schwellen dominieren, stellen eine Methode dar, die auch eine besonders hohe Test-Retest-Zuverlässigkeit aufweist [9, 20, 23]. Wenn man aber berücksichtigt, dass die Hörschwelle etwa im Verhältnis 1:2 mit den DPOAE-Pegeln zusammenhängt [18], dann lassen sich signifikante Unterschiede in der Test-Retest-Zuverlässigkeit von DPOAE-Pegeln und Schwellen – sowohl DPOAE-Schwellen als auch Hörschwellen – nur abschätzen, nachdem die DPOAE-Pegel zunächst mit zwei multipliziert wurden [3]. Bei dem Vergleich der Test-Retest-Zuverlässigkeit muss darüber hinaus immer berücksichtigt werden, wieviel Messzeit aufgewandt wurde, aber auch, dass DPOAE-Pegel primär Informationen über das überschwellige Verhalten des cochleären Verstärkers beinhalten, während DPOAE-Schwellen eher das Verhalten nahe der neuronalen Schwelle und damit die maximale cochleäre Verstärkung charakterisieren.

Ziel der vorliegenden Studie ist es, mithilfe eines kombinierten Analyseparadigmas aus Reintonhörschwelle (*L*_TA_), „estimated distortion-product threshold“ (*L*_EDPT_) und DPOAE-Pegel (*L*_DP_) den Einfluss von Messungenauigkeiten der jeweiligen Methoden zu reduzieren und die Test-Retest-Zuverlässigkeit zu erhöhen.

## Studiendesign und Untersuchungsmethoden

### Studiendesign und Messsystem

Für das hier vorgestellte kombinierte Analyseparadigma wurden DPOAE-Pegelkarten und Hörschwellen aus einer von den Autoren durchgeführten Studie [3] verwendet, in der die Test-Retest-Zuverlässigkeit von pegelkartenbasierten *L*_EDPT_ mit der von Reintonschwellen verglichen wurde. Die Messungen wurden siebenmal innerhalb von drei Monaten bei 14 Frequenzen zwischen 1 und 14 kHz in 20 Ohren von zehn normalhörenden Personen (PTA_4 (0,5–4_ _kHz)_ < 20 dB HL; Alter 32,1 ± 9,7 J.) aufgezeichnet. Die subjektiven Hörschwellen, *L*_TA_, wurden mittels modifizierter Békésy-Tracking-Audiometrie dreimal bei jeder Frequenz und bei zwei benachbarten Frequenzen erfasst, wodurch eine Frequenzgruppe gebildet wurde, um Feinstruktureffekte im Verhaltensaudiogramm zu glätten. Die Studie wurde von der Ethikkommission der Universität Tübingen genehmigt (265/2018B01) und in Übereinstimmung mit der Deklaration von Helsinki für Experimente am Menschen durchgeführt.

Alle Messungen wurden mit zwei über NI-Messkarten (National Instruments, Austin, TX, USA) an einen handelsüblichen PC angeschlossenen ER-10C-Messsonden (Etymotic Research, Elk Grove Village, IL, USA) durchgeführt. Stimulation und Messdatenerfassung erfolgten mit einer in LabVIEW (NI, Austin, TX, USA) implementierten Messsoftware. Eine eigens entwickelte Software in MATLAB (The MathWorks, Natick, MA, USA) ermöglichte eine automatisierte Analyse der DPOAE und Hörschwellen. Um eine möglichst gleiche Position der Messsonden über alle Sitzungen hinweg zu erreichen, wurde der Frequenzgang des Gehörgangsschalldrucks je Ohr von 0,3 bis 20 kHz vor der Kalibrierung bestimmt und visuell mit dem der vorherigen Aufzeichnungen verglichen. Der Reizschalldruck wurde vor jeder Sitzung durch eine Im-Ohr-Messung kalibriert und die Übertragung zum Trommelfell mithilfe eines künstlichen Ohrs korrigiert (B&K Typ 4157, Brüel & Kjær, Nærum, Dänemark).

### DPOAE-Pegelkarten

**Abb. 1:**
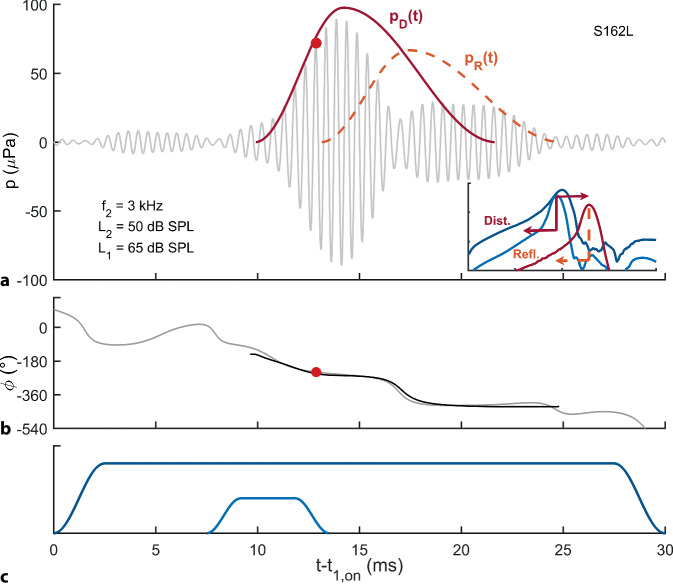
Trennung der DPOAE-Hauptkomponenten im Zeitbereich mithilfe der Kurzpuls-Stimulation. DPOAE werden mit zwei gepulsten Stimulustönen unterschiedlicher Länge der Frequenz *f*_2_ (*hellblau*) und *f*_1_ (*dunkelblau*) sowie den Stimuluspegeln *L*_2_ und *L*_1_ angeregt (**c**, schematische Darstellung). Entlang der ausgerollten Basilarmembran (Ausschnitt in **a**) werden die Einhüllenden der Wanderwellen skizziert (*Ordinate*: logarithmisch über drei Dekaden). In der Nähe des Abbildungsortes des *f*_2_-Tons entsteht infolge der Interaktion der *f*_2_- und *f*_1_-Wanderwellen die sog. nichtlineare Distorsionskomponente (*Dist.*) mit der Frequenz *f*_DP_ = 2*f*_1_ − *f*_2_, die sich u. a. auch direkt retrograd als Druckwelle in Richtung ovales Fenster ausbreitet. Zudem wirkt sie als intracochleärer Stimulus und erzeugt eine weitere Wanderwelle (*dunkelrot*), die sich anterograd zum Abbildungsort von *f*_DP_ ausbreitet. Dort wird ein Teil der Wanderwelle an lokalen Streuzentren reflektiert und breitet sich ebenfalls in Richtung des ovalen Fensters aus und bildet damit die Reflexionskomponente (*Refl.*). Zeitverlauf der DPOAE-Amplitude (**a**) und Phase (**b**) relativ zum Anschaltzeitpunkt *t*_1,on_ des *f*_1_-Tones, aufgezeichnet im Ohr S162L bei *f*_2_ = 3 kHz, *L*_2_ = 50 dB SPL, *L*_1_ = 65 dB SPL. **a** Gemessenes DPOAE-Signal (*hellgraue Linie*). *Dunkelroter Punkt*: Amplitude der nichtlinearen Distorsionskomponente *P*_DP_. Einhüllende der Distorsionskomponente, *p*_D_(*t*) (*dunkelrote Linie*), und Reflexionskomponente, *p*_R_(*t*) (*hellrote gestrichelte Linie*). **b** Instantane Phase des gemessenen DPOAE-Signals (*hellgrau*) und instantane Phase des berechneten DPOAE-Signals, *p*_D_(*t*) + *p*_R_(*t*) (*schwarz*). DPOAE bestehen im Wesentlichen aus zwei Komponenten, die an zwei verschiedenen Orten in der Cochlea durch zwei unterschiedliche Mechanismen entstehen. In Abhängigkeit von den relativen Amplituden und Phasen zwischen den Komponenten interferieren die Wellen, und können sich beispielsweise wie hier im Zeitsignal (**a**) gezeigt bei einem Phasenunterschied von knapp 180° (**b**) annähernd auslöschen und damit eine Schädigung vortäuschen, wenn keine Komponententrennung erfolgt. Da die DPOAE-Komponenten unterschiedliche Latenzen aufweisen, können die beiden DPOAE-Komponenten mithilfe der Kurzpuls-Stimulation und der Darstellung im Zeitbereich voneinander getrennt werden. Für eine ausführliche Illustration der Kurzpuls-Stimulation möchten wir den interessierten Leser gerne auf Zelle et al. (2016) [29] verweisen

Für die bilaterale Erfassung von DPOAE-Pegelkarten wurden je 21 Kurzpuls-DPOAE unterschiedlicher Anregungspegel (*L*_1,_*L*_2_-Paare) bei einem Frequenzpaar (*f*_2_ = 1–14 kHz, *f*_2_/*f*_1_ = 1,2) erfasst. Die Kurzpuls-Stimulation ermöglicht die Trennung der nichtlinearen Distorsions- und kohärenten Reflexionskomponenten im Zeitbereich durch Ausnutzung ihrer unterschiedlichen Latenz (Abb. 1).

Die aus dem Zeitsignal extrahierte artefaktfreie DPOAE-Amplitude, *P*_DP_ (roter Punkt in Abb. 1a), die die nichtlineare Distorsionskomponente bewertet, wurde bei einem SNR ≥ 10 dB für die statistische Analyse akzeptiert und in den DPOAE-Pegel, *L*_DP_, umgerechnet. Die Gesamtmesszeit für alle DPOAE-Pegelkarten mit 21 Pegeln bei 14 Frequenzen in beiden Ohren betrug 12,6 min.

**Abb. 2:**
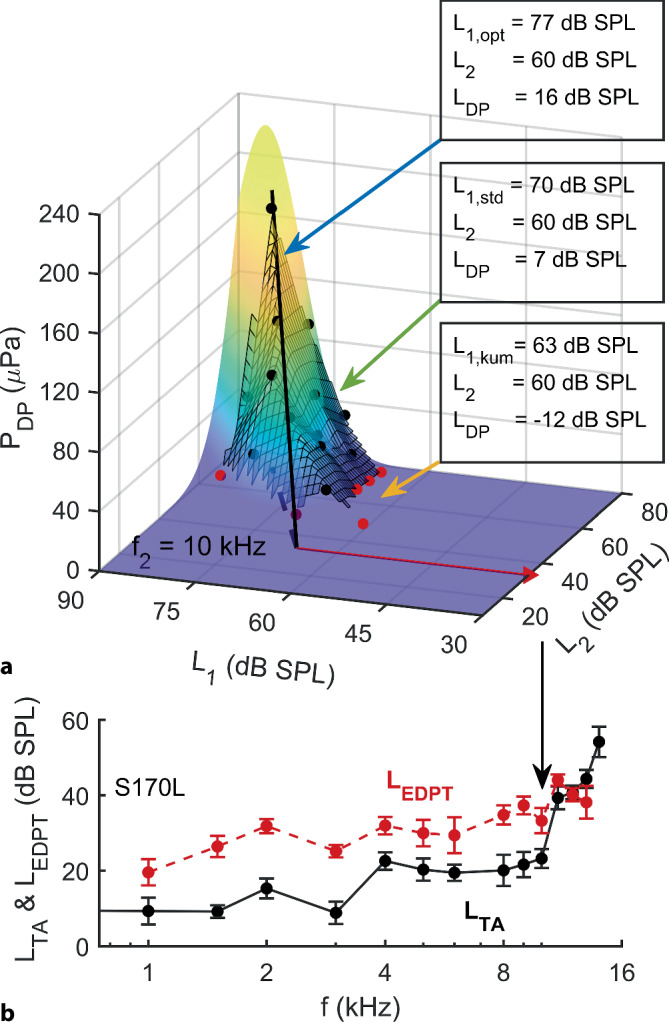
**a** Individuelle Modellpegelkarte, rekonstruiert aus akzeptierten DPOAE-Amplituden *P*_DP_ (*schwarze Punkte*), aufgezeichnet am Ohr S170L bei *f*_2_ = 10 kHz. *Rote Punkte*: *P*_DP_ mit SNR < 10 dB. *Schwarze durchgezogene Linie*: Grat, der die optimale, semi-logarithmische Wachstumsfunktion darstellt. *Roter Pfeil*: Geschätzte Distorsionsproduktschwelle *L*_EDPT_ mithilfe der DPOAE-Pegelkarte; *L*_EPDT_ = 33,25 ± 3,34 dB SPL. In diesem Beispielohr, bei *L*_2_ = 60 dB SPL, wären der als standard (std) klassifizierte Anregungspegel *L*_1,std_ = 70 dB SPL (*grüner Pfeil*) sowie der nach Kummer et al. (kum) gewählte Anregungspegel *L*_1,kum_ = 63 dB SPL (*gelber Pfeil*) suboptimal und würden deutlich verringerte *P*_DP_ bzw. DPOAE-Pegel (*L*_DP_) im Vergleich zu dem individuell optimalen (opt) Anregungspegel *L*_1,opt_ = 77 dB SPL (*blauer Pfeil*) generieren. **b** EDPT-Gramm *L*_EDPT_ (*rot*) und das Audiogramm *L*_TA_ (*schwarz*) für *f* = 1–14 kHz. Der Verlauf des EDPT-Gramms zeigt eine hohe Korrelation mit dem subjektiven Tonschwellenaudiogramm

Die dreidimensionale Darstellung des DPOAE-Wachstumsverhaltens in Abhängigkeit der Anregungspegel *L*_1_ und *L*_2_ ergibt DPOAE-Pegelkarten (Abb. 2), die mithilfe einer numerischen Anpassung einer mathematischen Funktion an die Messwerte die Rekonstruktion einer individuell optimalen Wachstumsfunktion (Abb. 2, schwarze Linie) und somit die Berechnung von *L*_EDPT_ (Abb. 2, roter Pfeil) für jede Frequenz ermöglichen. Aus der Projektion der optimalen Wachstumsfunktion in der *L*_1_,*L*_2_-Ebene können für jeden *L*_2_-Pegel die individuell optimalen (opt) frequenzspezifischen Anregungspegel *L*_1,opt_ ermittelt werden, die jeweils maximale *P*_DP_ generieren. Mit dieser Methode können *L*_EDPT_ bestimmt werden, ohne dass optimale Anregungspegel vorab definiert werden müssen [28]. Zudem können anhand der Modellpegelkarten diejenigen *L*_DP_ rekonstruiert werden, die mit einem frequenzunabhängigen Anregungsparadigma nach Kummer et al. (1998) [18], *L*_1,kum_ = 0,4 *L*_2_ + 39 dB, oder mit einem konstanten als standard (std) klassifizierten Pegelabstand, *L*_1,std_ = *L*_2_ + 10 dB, generiert worden wären. In der vorliegenden Arbeit erfolgten diese Berechnungen exemplarisch für *L*_2_ = 45 und 65 dB SPL. Man muss sich bewusst sein, dass diese rekonstruierten *L*_DP_ nur insoweit sinnvolle Abschätzungen über tatsächliche Messwerte liefern, wie die Modellfunktion die tatsächliche Abhängigkeit von den Anregungspegelkombinationen getreu nachbildet. Sowohl deswegen als auch wegen üblicher Grenzbedingungen der praktischen Messbarkeit (i. d. R. Pegel des Restrauschens < −20 dB SPL [4, 12]) wurden rekonstruierte *L*_DP_ < −15 dB SPL nicht gewertet und aus den weiteren Analysen ausgeschlossen.

### Kombinierte Analyse

Für eine kombinierte Betrachtung zeitgleich auftretender Veränderung in *L*_DP_ und *L*_EDPT_ bzw. *L*_TA_ innerhalb eines Ohres von Untersuchung zu Untersuchung wurde die Korrelation der einzelnen DPOAE-Metriken mit der Reintonhörschwelle ermittelt. *L*_EDPT_ korrelieren mit *L*_TA_ ungefähr im Verhältnis 1:1 [28, 30], d. h. eine Erhöhung der *L*_TA_ um 10 dB geht mit einer Erhöhung der *L*_EDPT_ um ebenfalls etwa 10 dB einher. Die Schätzung der Korrelation der DPOAE-Pegel mit der Reintonhörschwelle erfolgte anhand der Studie von Kummer et al. (1998) [18] , s. deren Abb. 7b bzw. deren Tab. II, III, wobei eine Erhöhung der *L*_TA_ um 10 dB ungefähr mit einer Abnahme der *L*_DP_ um 5 dB verbunden ist, also in einem Verhältnis von ca. 2:1 korreliert. In der Gesamtschau bedeutet dies, dass, um aus einer *L*_DP_-Abnahme eine cochleär bedingte Hörschwellenerhöhung abzuschätzen, die entsprechende *L*_DP_-Abnahme verzweifacht werden müsste. Somit wurden Hörschwellenänderungen mit den doppelten entgegengerichteten *L*_DP_-Änderungen für eine kombinierte Analyse in unterschiedlichen Kombinationen gemittelt, d. h. (∆*L*_TA_ − 2∆*L*_DP_)/2, (∆*L*_TA_ + ∆*L*_EDPT_)/2, (∆*L*_EDPT_ − 2∆*L*_DP_)/2 und (∆*L*_TA_ + ∆*L*_EDPT_ − 2∆*L*_DP_)/3.

### Statistische Auswertung

Für die statistischen Tests wurde SPSS Statistics (Version 26, Fa. IBM Corp., Armonk, NY, USA) verwendet. Zur Quantifizierung der Test-Retest-Zuverlässigkeit der *L*_EDPT_, *L*_TA_ und *L*_DP_ wurden die absoluten Differenzen (AD) zwischen zwei Visiten (1 vs. 2, 1 vs. 3, 1 vs. 4, …, 2 vs. 3, 2 vs. 4, …; *N* = 21) als Metrik verwendet [20, 21]. Die Test-Retest-Zuverlässigkeit bestimmt die Fähigkeit einer Methode, ähnliche Ergebnisse zu liefern, wenn sie für dieselbe Versuchsperson unter denselben Versuchsbedingungen wiederholt wird. Die statistische Signifikanz der absoluten Unterschiede zwischen den Stichproben wurde mit dem Friedman-Test geprüft.

## Ergebnisse

### DPOAE-Pegel

**Abb. 3:**
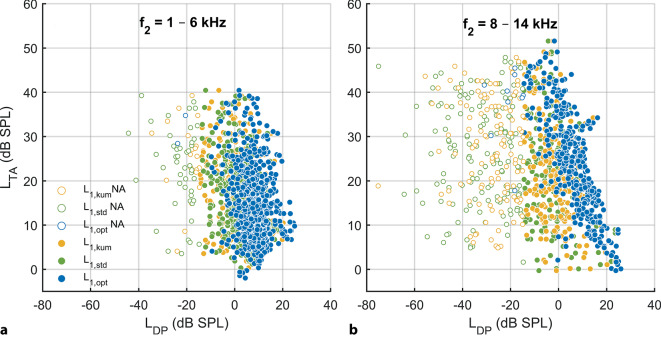
Korrelation der Hörschwelle (*L*_TA_ in dB SPL, erfasst mithilfe der modifizierten Békésy-Tracking-Audiometrie) mit den DPOAE-Pegel (*L*_DP_ in dB SPL, rekonstruiert mithilfe der individuellen DPOAE-Modellpegelkarte). Die Anregungspegel wurden folgendermaßen gewählt: *L*_2_ = 45 dB SPL, *L*_1_ entsprechend modifiziert. *L*_1,opt_: individuell optimale, frequenzspezifische Anregungspegel (*blau*), *L*_1,std_ = *L*_2_ + 10 dB (*grün*) und *L*_1,kum_ = 0,4 *L*_2_ + 39 dB (*gelb*). *L*_DP_ ≤ −15 dB SPL werden als nicht akzeptiert gewertet und durch offene *Kreise* dargestellt. **a** Frequenzbereich *f*_2_ = 1–6 kHz, *N* = 649/980. **b** Frequenzbereich *f*_2_ = 8–14 kHz, *N* = 353/980

Die Korrelation zwischen *L*_DP_ und *L*_TA_ hing wesentlich von der Wahl der Anregungspegel ab (Abb. 3). Für den Frequenzbereich *f*_2_ = 8–14 kHz, in welchem bereits bei einigen Probanden ein Hochtonhörverlust vorlag und damit ein größerer Dynamikbereich, korrelierten *L*_DP_ und *L*_TA_ stark miteinander (Spearman-Rho, *ρ* = −0,737; *p* < 0,001), wenn *L*_DP_ mithilfe von individuell optimalen, frequenzspezifischen Anregungspegeln *L*_1,opt_ (blau) bei *L*_2_ = 45 dB SPL rekonstruiert wurde (Tab. 1). Wenn *L*_DP_ dagegen mit nichtfrequenzspezifischen, klinisch häufig verwendeten Anregungspegeln *L*_1,std_ (grün, *L*_1_ = *L*_2_ + 10 dB) bzw. *L*_1,kum_ (gelb, *L*_1_ = 0,4*L*_2_ + 39 dB) rekonstruiert wurde, korrelierten *L*_DP_ und *L*_TA_ nur noch gering (*L*_1,std_ Spearman-*ρ* = −0,202; *p* = 0,003; *L*_1,kum_ Spearman-*ρ* = −0,282; *p* < 0,001). Für den Frequenzbereich *f*_2_ = 1–6 kHz lag wenig Hörverlust und daher kein ausreichender Dynamikbereich vor, um damit die Korrelation zwischen *L*_DP_ und *L*_TA_ abschließend zu beurteilen. Der Anregungspegel *L*_2_ = 45 dB SPL wurde exemplarisch dargestellt, da bei diesem Pegel der cochleäre Verstärker noch nicht in Kompression ist und eine ausreichende Anzahl von nachweisbaren DPOAE-Signalen generiert wurden.

**Tab. 1:** Lineare Regressionsanalyse der abhängigen Variable Hörschwelle *L*_TA_ (dB SPL) von der unabhängigen Variable DPOAE-Pegel *L*_DP_ (dB SPL) für die unterschiedlichen Anregungsparadigma und Frequenzbereiche (*L*_2_ = 45 dB SPL); Daten sind in Abb. 3 gezeigt. Die Güte der Schätzung, quantifiziert durch die Standardabweichung σ (dB) der Schätzung, ist für *f*_2_ = 8–14 kHz deutlich höher für *L*_1,opt_ im Vergleich zu *L*_1,std_ und *L*_1,kum_. *r*^*2*^: Korrelationskoeffizient zum Quadrat

*f*_2_		y =	*r*^2^	σ	*p*	*N*
1–6 kHz	*L*_1,opt_	16,57 – 0,268*x*	0,030	8,05	< 0,001	649
	*L*_1,kum_	16,57 – 0,479*x*	0,136	7,43	< 0,001	631
	*L*_1,std_	15,81 – 0,484*x*	0,138	7,24	< 0,001	605
8–14 kHz	*L*_1,opt_	30,62 – 1,079*x*	0,601	7,13	< 0,001	353
	*L*_1,kum_	22,41 – 0,479*x*	0,125	10,38	< 0,001	240
	*L*_1,std_	21,77 – 0,358*x*	0,071	10,69	< 0,001	215

**Abb. 4:**
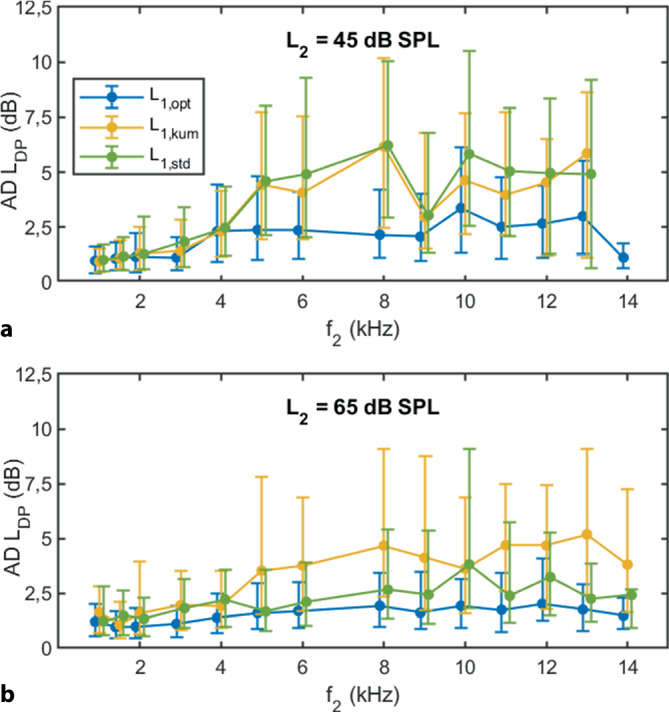
Test-Retest-Zuverlässigkeit der DPOAE-Pegel *L*_DP_ dargestellt mithilfe des Medians der absoluten Differenzen (AD) für die einzelnen Frequenzen in Abhängigkeit von den Anregungspegeln *L*_1,opt_ (*blau*), *L*_1,kum_ = 0,4L_2_ + 39 dB (*gelb*) und *L*_1,std_ = *L*_2_ + 10 dB (*grün*). Der *untere* Fehlerbalken entspricht dem 25. Perzentil, der *obere* Fehlerbalken dem 75. Perzentil. Die Graphen wurden entlang der Abszisse für die bessere Lesbarkeit leicht versetzt. **a** *L*_2_ = 45 dB SPL. **b** *L*_2_ = 65 dB SPL

Die Test-Retest-Zuverlässigkeit von *L*_DP_ erhöhte sich signifikant mit individuell optimalen Anregungspegeln (*L*_1,opt_; Abb. 4). Der Median von AD von *L*_DP_ verringerte sich signifikant von 2,3 bzw. 2,2 dB auf 1,8 dB mithilfe *L*_1,opt_ bei *L*_2_ = 45 dB SPL (Tab. 2, Friedman-Test, *F* = 734,65; *p* < 0,0001), und damit verringerte sich das daraus abgeleitete Referenzintervall von 10 bzw. 9 dB auf 6 dB, hier als 90. Perzentile der AD definiert. Bei *L*_2_ = 65 dB SPL reduzierte sich der Median der AD mithilfe *L*_1,opt_ von 2,4 bzw. 1,9 dB auf 1,4 dB und das Referenzintervall von 9 bzw. 7 dB auf 4 dB (Tab. 2).

**Tab. 2:** Test-Retest-Zuverlässigkeit der DPOAE-Pegel *L*_DP_ in Abhängigkeit von den Anregungspegeln für *f*_2_ = 1–14 kHz, dargestellt mithilfe des Medians, des Interquartilsabstands (IQR) und des 90. Perzentils der absoluten Differenzen (dB) für *L*_2_ = 45 und 65 dB SPL, und jeweils *L*_1,opt_, *L*_1,kum_ = 0,4*L*_2_ + 39 dB und *L*_1,std_ = *L*_2_ + 10 dB. Die Test-Retest-Zuverlässigkeit von *L*_DP_ erhöhte sich signifikant durch die Wahl individuell optimaler, frequenzspezifischer Anregungspegel *L*_1,opt_ bei *L*_2_ = 45 und 65 dB SPL (Friedman-Test, *F* = 383,90/482,37; *p* < 0,0001)

		Absolute Differenzen DPOAE-Pegel (AD *L*_DP_)						
	*L*_2_ = 45 dB SPL				*L*_2_ = 65 dB SPL			
	Median	IQR	90. Perzentil	*N*	Median	IQR	90. Perzentil	*N*
*L*_1,opt_	1,8	2,8	6,2	2469	1,4	1,9	4,1	2508
*L*_1,kum_	2,2	4,0	8,9	2123	2,4	4,2	9,4	2345
*L*_1,std_	2,3	4,3	9,5	1991	1,9	2,9	7,0	2499

### Geschätzte Hörschwellen anhand von DPOAE-Pegelkarten

**Abb. 5:**
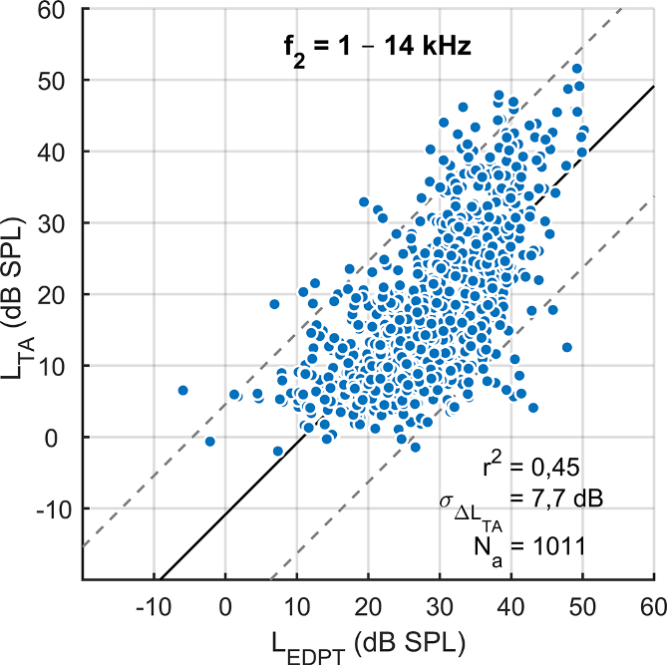
Korrelation der subjektiven Hörschwellen *L*_TA_, erfasst mithilfe der modifizierten Békésy-Tracking Audiometrie, mit den auf DPOAE basierenden Hörschwellenschätzungen *L*_EDPT_, abgeleitet von DPOAE-Pegelkarten; gepoolt wurde über alle Frequenzen, Probanden und Visiten. *Schwarze durchgezogene Linie* und *graue gestrichelte Linien* stellen die Regressionslinie bzw. 95%-Konfidenzintervalle dar. Standardabweichung (SD) der *L*_TA_ von der Regressionsgeraden $\sigma _{{\Updelta L_{\mathrm{TA}}}}$ = 7,7 dB. Anzahl der akzeptierten *L*_EDPT_ *N*_a_ = 1011/1960

**Abb. 6:**
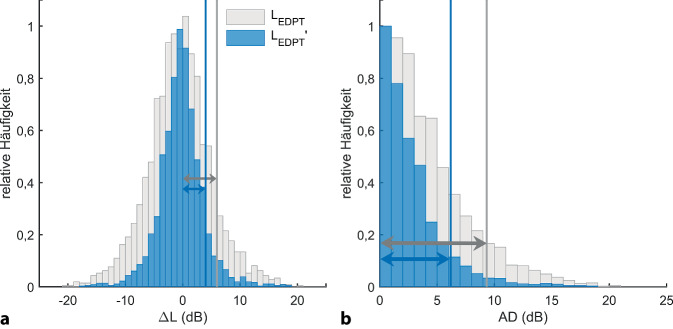
**a** Test-Retest-Zuverlässigkeit einer alleinigen DPOAE-Pegelkarten-basierten Hörschwellenschätzung *L*_EDPT_ im Vergleich zu dem kombinierten Maß *L*_EDPT_′ = (∆*L*_EDPT_ + ∆*L*_TA_ − 2∆*L*_DP,opt,65_)/3, dargestellt mithilfe der normierten Histogramme der Differenzen zwischen zwei Untersuchungen, gepoolt über alle Probanden und Frequenzen *f*_2_ = 1–14 kHz. *N* = 2345/5880. *L*_EDPT_′ zeigt im Vergleich zu den *L*_EDPT_ eine deutliche Reduktion der Ausreißer sowie der Standardabweichung von 5,6 dB (*grauer Pfeil*) auf 3,9 dB (*blauer Pfeil*). **b** Histogramme der absoluten Differenzen (AD) von *L*_EDPT_ (*grau*) und *L*_EDPT_′ (*blau*). Die *vertikalen Linien* markieren den Referenzbereich, definiert durch das 90. Perzentil. Mithilfe des kombinierten Maßes reduziert sich der Referenzbereich von 9,3 auf 6,2 dB, und damit wären tatsächliche Veränderungen der Hörschwelle deutlich früher detektierbar

*L*_EDPT_ und *L*_TA_ zeigen eine lineare Korrelation (*L*_TA_ = 0,86*L*_EDPT_ − 6,7 dB; *r*^2^ = 0,45; SD = 7,7 dB; *p* < 0,001; Abb. 5). Neben der hohen Korrelation mit der Reintonhörschwelle zeigten *L*_EDPT_ zudem eine hohe Test-Retest-Zuverlässigkeit mit einem Median der AD von 3,3 dB für *f*_2_ = 1–14 kHz (Tab. 3). Die Tab. 3 quantifiziert die Test-Retest-Zuverlässigkeit mithilfe des Medians, des Interquartilsabstands (IQR) und des 90. Perzentils für die alleinige Hörschwellenerfassung bzw. -schätzung mit *L*_TA_ und *L*_EDPT_ sowie für unterschiedliche kombinierte Analyseparadigma. Die Kombination von *L*_EDPT_, *L*_TA_ und *L*_DP,opt,65_ (bei *L*_2_ = 65 dB SPL und *L*_1,opt_) zeigte die kleinste AD. Eine fast ebenso niedrige AD lässt sich feststellen, wenn beide DPOAE-Maße, also *L*_DP_ und *L*_EDPT_, kombiniert wurden. In Abb. 6a werden die Differenzen zwischen den einzelnen Untersuchungen mithilfe von Histogrammen dargestellt, dabei zeigt sich für das kombinierte Maß aus *L*_EDPT_, *L*_TA_ und *L*_DP,opt,65_, bezeichnet als *L*_EDPT_′, im Vergleich zu den *L*_EDPT_ eine deutliche Reduzierung der Ausreißer sowie eine Verringerung der Standardabweichung von 5,6 dB auf 3,9 dB (vertikale Linien). In Abb. 6b werden die absoluten Differenzen (AD) zwischen den einzelnen *L*_EDPT_ und *L*_EDPT_′ mithilfe von Histogrammen dargestellt. Die vertikalen Linien markieren den Referenzbereich, definiert durch das 90. Perzentil. Mithilfe des kombinierten Maßes reduziert sich der Referenzbereich von 9,3 auf 6,2 dB. Zusammengenommen bedeuten diese Beobachtungen, dass das kombinierte Maß *L*_EDPT_′ eine deutlich höhere Test-Retest-Zuverlässigkeit aufweist als *L*_EDPT_.

**Tab. 3:** Test-Retest-Zuverlässigkeit der Hörschwellen (*L*_TA_) mithilfe der modifizierten Békésy-Tracking Audiometrie, der DPOAE-basierten geschätzten Hörschwellen (*L*_EDPT_) und der kombinierten Maße für den Frequenzbereich *f*_2_ = 1–14 kHz, dargestellt mithilfe des Medians, IQR und der 90. Perzentile der absoluten Differenzen (AD). *L*_DP,opt,65_: *L*_DP_ bei *L*_1,opt_ für *L*_2_ = 65 dB SPL. Die Test-Retest-Zuverlässigkeit lässt sich durch die kombinierten Maße signifikant verringern (Friedman-Test, *F* = 788,52, *p* < 0,0001)

Maß	AD (dB)			
	Median	IQR	90. Perzentile	*N*
∆*L*_TA_	3,2	4,7	10,2	5607
∆*L*_EDPT_	3,3	4,4	9,3	2508
(∆*L*_EDPT_ + ∆*L*_TA_) / 2	2,5	3,5	7,6	2503
(∆*L*_TA_ − 2∆*L*_DP,opt,65_) / 2	2,3	3,1	7,1	2503
(∆*L*_EDPT_ − 2∆*L*_DP,opt,65_) / 2	2,1	3,1	6,3	2508
(∆*L*_EDPT_ + ∆*L*_TA_ − 2∆*L*_DP,opt,65_) / 3	2,0	2,9	6,2	2503

## Diskussion

### DPOAE-Pegel: Aussagekraft und Test-Retest-Zuverlässigkeit

In der Regel zeigen DPOAE-Pegel eine relativ begrenzte Korrelation mit dem cochleär induzierten Hörverlust, und die komplexe Beziehung zwischen DPOAE-Pegel und dem daraus resultierenden Hörverlust ist von Anregungspegel und -frequenz nichtlinear abhängig [4, 12]. Interessanterweise fand sich hier nach Berücksichtigung der individuell optimalen Anregungspegel *L*_1_ bei einem moderaten Anregungspegel *L*_2_ = 45 dB SPL vor allem im hochfrequenten Bereich *f*_2_ = 8–14 kHz eine deutlich höhere Korrelation von *L*_DP_ mit *L*_TA_ und eine geringere Streuung (Abb. 3). Da das Einschlusskriterium für die Studie mit dem PTA_4 (0,5–4_ _kHz)_ < 20 dB HL definiert wurde, lag für einzelne Frequenzen zwischen 1–6 kHz kaum Hörverlust und daher kein großer Dynamikbereich vor, um damit eine potenzielle Korrelation zwischen *L*_DP_ mit *L*_TA_ sowie eventuell damit verbundene Mittelohr- oder Rauscheinflüsse abschließend zu beurteilen.

Die Idee hinter der Verwendung individuell optimaler Anregungspegel ist es, eine möglichst ideale Überlappung zwischen den Wanderwellen der beiden Anregungstöne *f*_1_ und *f*_2_ am Abbildungsort des zweiten Anregungstons zu erreichen bzw. die unterschiedlichen Kompressionsraten der beiden Anregungstöne am Entstehungsort der nichtlinearen Distorsionskomponente zu berücksichtigen [24], sodass ein optimaler Modulationskontrast der nichtlinearen mechanoelektrischen Transduktion eine maximale Distorsion hervorruft [2]. Die Berücksichtigung dessen durch Rekonstruktion von *L*_DP_ bei individuell optimalen Anregungspegeln führte in dieser Studie zu einer Reduktion der interindividuellen Variabilität von *L*_DP_, insbesondere für *f*_2_ = 8–14 kHz (Abb. 3b).

Nicht nur die interindividuelle Variabilität von *L*_DP_, sondern auch die intraindividuelle Variabilität, die sog. Test-Retest-Zuverlässigkeit, verbesserte sich durch die Wahl frequenzspezifischer, individuell optimaler Anregungspegel deutlich. Damit ließe sich der häufig zitierte Referenzbereich für eine DPOAE-Veränderung von Untersuchung zu Untersuchung innerhalb eines Subjekts beim Anregungspegel von *L*_2_ = 65 dB SPL von ca. 6–8 dB auf 4–5 dB reduzieren. In zukünftigen klinischen Untersuchungen könnten daher gepulste DPOAE-Pegel angeregt mithilfe frequenzspezifischer, individuell optimaler Anregungspegel eine wertvolle Methode sein, um frühzeitig Anzeichen von Veränderungen des cochleären Verstärkers zu erkennen, bevor sie mit konventionellen DPOAE-Pegel-Messungen sichtbar werden.

### Geschätzte DPOAE-Schwellen: Aussagekraft und Test-Retest-Zuverlässigkeit

DPOAE-Pegelkarten bilden das Wachstumsverhalten des cochleären Verstärkers für unterschiedliche Anregungspegelpaare *L*_1_,*L*_2_ bei einer Frequenz *f*_2_ hochgenau ab. Daraus bestimmte *L*_EDPT_ inkorporieren Informationen multipler DPOAE-Amplituden und erlauben damit eine präzisere und erweiterte Diagnostik über die Funktionsfähigkeit des cochleären Verstärkers, wie es auch von uns und anderen Autoren bereits für Wachstumsfunktionen und deren Eigenschaften gezeigt werden konnte [26]. Anhand der numerischen Extrapolation der einzelnen DPOAE-Amplituden kann das Wachstumsverhalten der DPOAE-Amplituden auch bei niedrigen Anregungspegeln abgeleitet werden. Gerade dann ist die Aussagekraft über die Funktionsfähigkeit des cochleären Verstärkers aufgrund seiner Nichtlinearität und der maximalen Verstärkungsleistung bei niedrigen Anregungspegeln am größten [18].

Darüber hinaus erlauben *L*_EDPT_ eine objektive Quantifizierung der Hörschwelle im Vergleich zur klinisch gebräuchlichen Erfassung einzelner DPOAE-Pegel, wenn keine Schädigung der inneren Haarsinneszellen oder der neuralen Weiterleitung vorliegt [8, 28, 30]. Die individuellen Übertragungseigenschaften des Mittelohrs werden durch DPOAE-Pegelkarten berücksichtigt, indem sie die Verschiebung der intracochleären Anregungsstimuli durch anterograde Übertragungsverluste in *L*_1_ und *L*_2_ und die retrograden Mittelohrübertragungsverluste durch die DPOAE-Amplitude erfassen [19]. Die DPOAE-Wachstumsfunktion wird entlang des individuellen Grats extrapoliert (Abb. 2) und beruht somit auf maximalen DPOAE-Amplituden, die mithilfe individuell nahezu idealer Anregungspegel generiert wurden. *L*_EDPT_, die auf DPOAE-Pegelkarten basieren, schätzen Hörschwellen präziser im Vergleich zu herkömmlichen DPOAE-Wachstumsfunktionen, die mit vorgegebenen Stimuluspegeln angeregt werden [28]. In dieser Studie korrelierten *L*_EDPT_, basierend auf DPOAE-Pegelkarten, mit *L*_TA_ für *f*_2_ = 1–14 kHz mit einer Standardabweichung von 7,7 dB (Abb. 5). Dieser Wert ist im Vergleich zu der Studie von Zelle et al. [30] mit 6,5 dB deutlich höher, was auf die Reduktion der Mittelungszeit pro DPOAE auf ein Viertel und die Erweiterung des Frequenzspektrums von 1–8 kHz auf 1–14 kHz zurückgeführt wird. Zudem wurde bisher – aus Gründen der noch eingeschränkten Datenmenge – darauf verzichtet, die Korrelation frequenzabhängig durchzuführen, wodurch die Standardabweichung pro Frequenz deutlich verringert werden kann [30]. Insbesondere im hohen Frequenzbereich ist außerdem zu erwarten, dass die Implementierung eines modernen Kalibrierverfahrens wie beispielsweise IPL („integrated-pressure level“) oder FPL („forward-pressure level“) den Schätzfehler zwischen *L*_TA_ und *L*_EDPT_ weiter verringern würde [20].

*L*_EDPT_ schätzen nicht nur individuelle Hörschwellen präzise, sondern sind auch in Verlaufsuntersuchungen innerhalb eines Ohrs stabil [3]. Die Test-Retest-Zuverlässigkeit der *L*_EDPT_ ist für den gesamten Frequenzbereich *f*_2_ = 1–14 kHz mit dem Median der AD von 3,3 dB vergleichbar mit denen der *L*_TA_ (Median AD = 3,2 dB), wobei für den hochfrequenten Bereich, *f*_2_ = 11–14 kHz, *L*_EDPT_ gegenüber *L*_TA_ überlegen sind [3]. Der Referenzbereich entsprechend dem 90. Perzentil beträgt etwa 10 dB für *L*_EDPT_ und *L*_TA_ für *f*_2_ = 1–14 kHz, oberhalb dessen ein Ohr in Verlaufsuntersuchungen als kontrollbedürftig angesehen werden muss. *L*_DP_ weisen, wenn ihre Änderung für die geschätzte Abhängigkeit zur Hörschwelle durch Multiplizierung mit zwei korrigiert werden, eine vergleichbare Test-Retest-Zuverlässigkeit auf, nämlich mit einem Median der AD von 2,8–3,6 dB bzw. einem 90. Perzentil von 8–12 dB bei *L*_1,opt_ (Tab. 2). Da *L*_DP_ und *L*_EDPT_ teilweise unterschiedlichen Störfaktoren (z. B. Mittelohrpathologie, Rauschquellen) und physiologischen Mechanismen unterliegen, ist es naheliegend, die beiden unterschiedlichen Methoden zu kombinieren, um damit einen möglichst sensitiven und zuverlässigen Messparameter zu erhalten.

### Kombiniertes Analyseparadigma: Aussagekraft und Test-Retest-Zuverlässigkeit

Bisher werden im klinischen Alltag für das Monitoring von Ototoxizität i. d. R. Veränderungen der Reintonhörschwelle und DPOAE-Pegeländerung (meist bei *L*_2_ = 65 dB SPL, *L*_1,std_ = 75 dB SPL) isoliert betrachtet. Die kombinierte Betrachtung zeitgleich auftretender gleichgerichteter Hörschwellen- und DPOAE-Pegeländerungen wurde unseres Wissens nach bisher nicht angewandt oder in der Literatur beschrieben. Lediglich multivariate statistische DPOAE-Analysen, die DPOAE-Pegel und SNR zeitgleich betrachten, werden für eine potenzielle Vorhersage der Hörschwelle [11] und auch für eine potenzielle Vorhersage eines ototoxischen Hörschadens [16] dargestellt. Um den Hörstatus vorherzusagen, erreichen multivariate DPOAE-Analysen eine bessere Testgüte verglichen mit den univariaten Ansätzen, die entweder den DPOAE-Pegel oder den SNR verwenden. Allerdings gibt es selbst mit multivariaten Analysen noch erhebliche Überschneidungen zwischen den Verteilungen der Normalhörenden und Hörgeschädigten, die für den Frequenzbereich 0,75–3 kHz ausgeprägter als für 4–8 kHz ermittelt wurden [11]. Multivariate DPOAE-Analysen führen auch zu einer verbesserten Testgüte, um ototoxisch bedingte Hörschwellenerhöhungen vorherzusagen, allerdings nur, wenn die kumulative Cisplatin-Dosis in die Analyse einbezogen wird. Eine 6‑dB-DPOAE-Pegeländerung als Metrik gestattet wenig bis keine Verbesserung gegenüber einer Analyse, die auf der kumulativen Cisplatin-Dosis und der Präexpositionshörschwelle beruht [16]. Das hier vorgestellte kombinierte Analyseparadigma, das zeitgleich auftretende Veränderungen des cochleären Verstärkers in Verlaufsuntersuchungen mithilfe von *L*_EDPT_-, überschwelliger *L*_DP_-, und subjektiver feinstrukturreduzierter *L*_TA_-Änderung quantifiziert, verbesserte signifikant die Test-Retest-Zuverlässigkeit (Abb. 6 und Tab. 3). Es ist zu erwarten, dass damit in zukünftigen Untersuchungen eine höhere Sensitivität und Spezifität erzielt wird, um tatsächliche pathologische oder auch regenerative Veränderungen der äußeren Haarsinneszellen zu detektieren.

Da in dieser Studie das Augenmerk auf der Validierung der Methodik gepulster DPOAE in Verlaufsmessungen in normalhörenden Probanden lag, finden sich wenig Daten, die einen gering- bis mittelgradigen Hörverlust für *f*_2_ = 1–6 kHz widerspiegeln können, sodass die Korrelation der DPOAE-Pegel mit der Hörschwelle in einem Verhältnis von 1:2 primär anhand der Daten von Kummer et al. (1998) [18] überschlägig abgeschätzt wurde. Für zukünftige Anwendungen des kombinierten Analyseparadigmas sollten aufgrund der nichtlinearen Frequenz- und Pegelabhängigkeit der DPOAE-Pegel die Relation zwischen DPOAE-Pegel und Hörverlust mithilfe individuell optimaler Anregungspegel *L*_1_ frequenz- und pegelabhängig quantifiziert und sodann einbezogen werden.

Auch wenn hier gezeigt wurde, dass kombinierte Verfahren mit gepulsten DPOAE eine höhere Test-Retest-Zuverlässigkeit aufweisen als bisher aus der Literatur bekannt, ist noch nachzuweisen, dass bspw. eine ototoxisch bedingte Hörschädigung in Verlaufsuntersuchungen von Patienten, die eine Chemotherapie mit Cisplatin erhalten, durch Einsatz von DPOAE-Pegelkarten und kombinierten Auswerteverfahren frühzeitiger und sensitiver im Vergleich zu anderen audiologischen Testverfahren erfasst werden kann.

Darüber hinaus könnte das Verfahren durch weitere technische Anpassungen optimiert werden. Dies wäre zum einen die Implementierung eines modernen Kalibrierverfahrens, welches fehlerhafte Anregungspegel durch stehende Wellen innerhalb des Gehörgangs vermeidet und damit die Detektion der DPOAE in noch höherer Anzahl und Güte zulässt. Zudem wäre die Entwicklung eines adaptiven Messverfahrens von Vorteil, welches die Erfassung von DPOAE-Pegelkarten innerhalb eines *L*_1_,*L*_2_-Raums SNR-abhängig ermöglicht, um damit zeiteffizient bei möglichst jedem Patienten mit cochleärem Restgehör zuverlässig DPOAE-Pegelkarten zu erfassen.

## Ausblick

Objektive Hörschwellenschätzungen, basierend auf artefaktfreien Kurzpuls-DPOAE-Pegelkarten, sind aufgrund ihrer hohen Test-Retest-Zuverlässigkeit und der direkten Beziehung zur Funktionsfähigkeit des cochleären Verstärkers vielversprechend, um Hörverlust, z. B. bei Ototoxizität, frühzeitig und sensitiv zu detektieren. Sie erlauben eine einfache, zeiteffiziente Einordnung der Messergebnisse, da sie Hörschwellenschätzungen darstellen, die mit klinisch gebräuchlichen Hörschwellenbestimmungen wie der Reintonaudiometrie direkt vergleichbar sind. Die zeitgleiche Betrachtung von innerhalb eines Individuums auftretenden Veränderungen der DPOAE-Pegel sowie der subjektiv und objektiv bestimmten Hörschwellen mithilfe des hier vorgestellten kombinierten Analyseparadigmas reduziert den Einfluss von Messungenauigkeiten der jeweiligen einzelnen Verfahren. Somit könnten pathologische oder regenerative Veränderungen der Funktionsfähigkeit des cochleären Verstärkers deutlich früher als mit konventionellen Hörtests detektiert werden. Dies könnte frühere Interventionen und potenziell bessere Behandlungsergebnisse für Patienten ermöglichen.

## Fazit für die Praxis

- Konventionelle DPOAE-Verfahren in Verlaufsuntersuchungen erlauben bisher keine klinisch validierte Festlegung einer DPOAE-Änderung, die einen potenziellen Cochleaschaden wie z. B. bei Ototoxizität vorhersagen kann.
- In Verlaufsuntersuchungen hängen die Aussagekraft und die Test-Retest-Zuverlässigkeit der gepulsten DPOAE-Pegel entscheidend von der Wahl der Anregungspegel und ihrer Abweichung vom individuell optimalen Stimulus ab.
- DPOAE-Pegelkarten basierend auf gepulsten DPOAE ermöglichen eine präzise Hörschwellenschätzung und berücksichtigen dabei Interferenzeffekte der DPOAE-Komponenten sowie die individuelle Mittelohrübertragungsfunktion.
- Mithilfe eines kombinierten Analyseparadigmas ist es zu erwarten, dass die Zuverlässigkeit der Detektion einer Veränderung der Funktionsfähigkeit des cochleären Verstärkers im Vergleich zu konventionellen DPOAE-Verfahren deutlich erhöht werden kann.
